# Recognition of depression, anxiety, and alcohol abuse in a Chinese rural sample: a cross-sectional study

**DOI:** 10.1186/s12888-016-0802-0

**Published:** 2016-04-06

**Authors:** Yu Yu, Mi Hu, Zi-wei Liu, Hui-ming Liu, Joyce P. Yang, Liang Zhou, Shui-yuan Xiao

**Affiliations:** Department of Social Medicine and Health Management, School of Public Health, Central South University, Changsha, China; Department of Psychology, University of Washington, Seattle, USA

**Keywords:** Mental health literacy, Recognition, Mental disorders, Rural, Chinese

## Abstract

**Background:**

Under-utilization of mental health services is a global health issue. Recognition of mental disorders, as the first step to seeking help from professional sources, has been well studied in developed countries, yet little is known about the situation in rural areas of developing countries like China. The purpose of the study is to understand the recognition of depression, anxiety, and alcohol abuse and its predictive factors in a Chinese rural sample

**Methods:**

Face-to-face interviews were conducted on a representative rural adult sample in a cross-sectional study in China (*N* = 2052). Respondents were presented with three vignettes depicting depression, anxiety and alcohol abuse and asked to label the disorder and its cause to assess their recognition of the three mental disorders. They also completed the Patient Health Questionnaire (PHQ-9), the Generalized Anxiety Disorder Scale (GAD-7), and the Alcohol Use Disorders Identification Test (AUDIT) to assess their current mental health status.

**Results:**

The alcohol abuse vignette was more frequently attributed as a mental problem than the depression vignette and anxiety vignette. The correct labeling rate was 16.1 % in the depression vignette, 15.5 % in the anxiety vignette, and 58.2 % in the alcohol vignette. Higher education is the common and also strongest factor positively predicting the recognition of all three vignettes. Beyond that, being female is an independent predictor of correct recognition of alcohol abuse, while recognition of depression and anxiety were positively predicted by younger age.

**Conclusions:**

Lower recognition of depression and anxiety as compared to alcohol abuse confirms the importance and need to increase the public’s awareness and knowledge about common mental disorders. Recognition of common mental disorders could be improved through general public campaign and education, while paying attention to the unique predictive factors for each specific disorder and implement targeted intervention.

**Electronic supplementary material:**

The online version of this article (doi:10.1186/s12888-016-0802-0) contains supplementary material, which is available to authorized users.

## Background

Mental health is a significant public health concern across the world. In 2010, mental and substance use disorders accounted for 7.4 % of all disability-adjusted life years (DALYs) worldwide, among which depression, anxiety, and alcohol abuse accounted for 40.5, 14.6, and 9.6 %, respectively [1]. According to the World Health Organization, by 2020, a quarter of the world’s population will suffer from a mental disorder, and mental diseases are projected to account for 15 % of the global disease burden [2]. In China, an estimated 170 million adults are suffering from a mental disorder, accounting for 20 % of total disease burden [3]. Mental disorders are associated with high economic costs [4, 5], criminal activities [3], suicide rates [6], and overall mortality rates [7]. A recent meta-analysis estimated that 14.3 % of deaths worldwide, or approximately 8 million deaths each year, are attributable to mental disorders [7].

The huge economic and social cost of mental disorders is amplified by the under-utilization of mental health services worldwide. For example, a cross-sectional study conducted in seven European countries found that nearly half (48 %) of those needing mental healthcare did not receive any formal mental health services [8]. A 2007 National Survey of Mental Health and Wellbeing in Australia reported that almost two-thirds of those with mental disorders never seek any treatment [9], a figure similar to that in the US [10]. Another nationally representative study with a Norwegian sample of 65,648 respondents also found that 87 % of those with depression and 75 % with anxiety disorders had never sought any help [11]. The situation is even more dire in developing countries, where both lack of mental health resources and services utilization were reported in Asian populations, Black populations and Brazilian populations [12–16]. For instance, in China, over 90 % of those who meet criteria for a mental disorder never received any treatment [3].

A number of factors have been identified as barriers preventing people with mental disorders from seeking treatment, including shortage of infrastructure, lack of qualified doctors, financial hardship, limited access to mental healthcare providers, social stigma, and low mental health literacy [3, 17–21]. Among all the barriers to treatment seeking, mental health literacy has been one of the most researched areas with abundant evidence showing that reluctance to seeking treatment is largely the result of low mental health literacy. Of the various aspects of mental health literacy, recognition of mental disorders emerges as an initial and important factor, as it is the first step to seeking help from professional sources [10, 18]. Numerous studies have documented that inability to recognize mental disorders is associated with delay in and reduced likelihood of help-seeking [6, 17–19, 22, 23]. For example, one study conducted at an Australian specialist anxiety clinic reported that 60 % of patients attributed their delayed treatment seeking to “lack of knowledge” [23]. Furthermore, there is also growing evidence showing that early and correct recognition of mental disorders contributes to effective communication with health professionals, early treatment, and thus better long-term health outcomes for those with mental disorders [24–26].

In light of the significant role of recognition of mental disorders in effective mental health services utilization, it is essential to understand the public’s recognition of some common mental disorders and its predictive factors. A growing body of research has studied the recognition of different mental disorders among different populations with various results. For instance, the recognition rate of depression was 76 % in primary health care workers in India [27], 75 % in Australian adults [28], and 58 % among American adults [29], but was only 35 % in urban Chinese adults [30] and 25 % in Japanese adults [31], and even lower in a sample of undergraduates in Sri Lanka with a rate of only 17.4 % [32]. Recognition of anxiety was even lower, with a rate of 28 % for anxiety disorder in a sample of general pracitoner in Catalonia [33], 21 % for generalized anxiety disorder (GAD) in China [30], 16.6 % for panic disorder and 15.9 % for GAD in the US [10], and 9.2 % for social phobia in Australia [28]. Further investigation of the factors related to recognition of mental disorders has identified a number of various factors associated with lower recognition, including male gender, older age, lower education, negative attitude to psychopharmacology, not having a history of mental health treatment, and no previous contact with mentally ill people [10, 30, 34–38]. Since the majority of previous studies were conducted in developed countries [10, 17, 28, 34–36, 39] or urban areas in developing countries [30, 40], or health workers instead of general populations in rural areas of developing countries [27, 33], and mainly focused on one class of mental disorder [6, 10, 17, 35, 39] or simply comparing the recognition rate of several mental disorders without fully exploring its predictive factors [28, 34], more needs to be known about the recognition of various mental disorders and its predictive factors in general populations of rural areas in developing countries like China.

The aim of the present study is to fill in the knowledge gap by investigating the recognition of depression, anxiety, and alcohol abuse in a rural Chinese sample. The study involved face-to-face interviews in which we assessed respondents’ recognition of depression, anxiety or alcohol abuse based on a brief vignette describing symptoms of these disorders. Two aspects of recognition were examined: (1) Correctly attributing the disorder as a mental problem; (2) Correctly naming the disorder, followed by a multivariate logistic regression to explore factors predicting the correct recognition of the three mental disorders.

## Methods

### Participants

The target population was residents aged 18–60 who have lived in the rural areas of Liuyang County, Hunan Province for over 6 months. Sample size was calculated using the basic formula for a cross-sectional study from textbook: *N* = 400*(Q/P), *a* = 0.05, *Q* = 1-P. P is the estimated prevalence of recognition rate. According to past studies in China [30], we set P as 20 %, which produced a sample size of 1600. Considering the non-response rate and refusal rate based on previous studies, we further expanded our sample by 20 % and came to a theoretical sample size of 1920. A multistage cluster-sampling method was adopted to identify subjects. Two towns (Gaoping and Yongan) were randomly selected from 33 towns of Liuyang county, and then two administrative villages (merged by several naturalistic villages for the purpose of administrative management) were randomly selected from each town, followed by two naturalistic villages (formed by natural geographic environment) randomly selected from each administrative village, leading to a total sampling frame of 8 naturalistic villages. All adults in all households of the 8 villages were included as our final sampling frame, that is, 2158 residents. Inclusion criteria were: (1) aged 18 to 60, (2) living in the rural areas for more than half a year. Exclusion criteria were: (1) not living in the areas during the research period, (2) having difficulty in communication due to serious physical or mental illness, or cognitively impaired or actively psychotic. A final sample of 2052 residents was produced. Details about the sampling process and flowchart of subject enrollment have been published elsewhere [41, 42].

### Procedures

Ethics approval was granted by the Ethics Review Committee of the School of Public Health of Central South University. A team of 15 postgraduates from the School of Public Health of Central South University were recruited as interviewers. All interviewers have a background of public health and preventive medicine, and also received some basic training for psychology and psychiatry. All interviewers received a 2-day uniform formal training to conduct the interviews provided by a psychologist (MH) before the formal study. The training was composed of half lecturing and half practice of role plays. Interviewers visited each household and explained the purpose and process of the study to the participants. After providing written informed consent, each eligible respondent was invited to complete a series of questionnaires (see measures below) by face-to-face interviews. At the end of each interviewing day, a meeting was held to review the interviewing process, to check the quality of questionnaires, as well as to discuss problems emerged during the interviews. All questionnaires were double-checked by two quality control persons to ensure that there were no inconsistencies or missing items, or any logic errors, and then handed to one quality control person for final checking. All participants were reimbursed with some small gifts such as kitchen utensils ($2) in return for their participation.

### Instruments

#### PHQ-9

Symptoms of depression were measured using the Patient Health Questionnaire (PHQ-9), a nine-item screening tool based on criteria for depressive disorders in the Diagnostic and Statistical Manual of Mental Disorders (DSM-IV) [43]. Respondents are asked whether they have been bothered with 9 symptoms in the past two weeks on a 4-point Likert scale from 0 = “not at all,” to 3 = “nearly every day”. The total score ranges from 0 to 27, with scores of 5, 10, 15, and 20 representing cut-points for mild, moderate, moderately severe, and severe depression, respectively. A meta-analysis of the scoring method of PHQ-9 showed that a cut-off point of ≥ 10 has the best diagnostic performance and is thus used in the current study to differentiate people screening positive for depression versus those who do not [44]. The Chinese version of PHQ-9 has been well validated in multiple studies [45–47] and demonstrated a good internal consistency in the current study, with a Cronbach’s α coefficient of 0.81.

#### GAD-7

Symptoms of anxiety were measured using the Generalized Anxiety Disorder Scale (GAD-7), a 7-item self-report scale developed by Spitzer et al. [48] to assess symptoms and screen for general anxiety. Respondents are asked to choose how often they have been bothered by anxiety symptoms on a 4-point Likert scale from 0 = “not at all” to 3 = “nearly every day”. The total score ranges from 0 to 21, with a score of ≥10 representing the optimum cut-off point for screening positive for anxiety disorders [49]. The Chinese version of GAD-7 has been widely used and well validated in multiple studies [50, 51] and demonstrated good internal consistency in the current study, with a Cronbach’s α coefficient of 0.88.

#### AUDIT

Alcohol use disorders were measured by the alcohol use disorders identification test (AUDIT), a 10-item scale developed by the World Health Organization (WHO) [52] to identify hazardous and harmful drinking in diverse settings and multicultural populations [53]. All item scores range from 0 to 4 and the total score ranges from 0 to 40, with a score of ≥8 representing the optimum cut-point for hazardous drinking [52]. The Chinese version of AUDIT has been widely used and well validated in multiple studies [54] and demonstrated acceptable internal consistency in the current study, with a Cronbach’s α of 0.67.

#### Recognition of three vignettes

Recognition of depression, anxiety, and alcohol abuse were evaluated by three vignettes developed by two psychiatrists (LZ and SYX) drawing on clinical experience, published vignette studies, and cases used in past research [17, 28, 34, 55, 56], which were all written to satisfy the DSM-IV and ICD-10 diagnostic criteria. After each vignette, participants were presented with two multiple-choice questions asking “What do you think is wrong with the person?” and “What do you think is the primary cause of this problem?” with answer choice options assessing disease labeling and disease attribution. In order to make the western-based vignettes more culturally relevant for China, we created common and familiar scenes for each case such as with family, co-workers or neighbors, and used appropriate words to describe symptoms of each disorder. Each vignette has been pilot-tested and proved feasible for use among a rural population. Detailed information about the questions and optional answers can be seen in the Additional file 1.

### Data analysis

Data were analyzed using STATA software version 12.0. Scales and indices were tested for reliability. Percentage of respondents choosing each answer option for the two questions of each vignette were calculated and presented in Tables 1 and 2. A multivariate logistic regression analysis was carried out to examine the effect of demographic characteristics and psychological factors on correct recognition of depression, anxiety, and alcohol abuse (see Table 4).

**Table 1:** Socio-demographics and clinical characteristics of the sample (*N* = 2052) ^a^

Characteristics	Number	Percent
Gender		
Male	907	44.20
Female	1145	55.80
Age (years)		
18–25	227	11.06
26–35	344	16.76
36–45	687	33.48
46–60	794	38.69
Education		
Primary school or lower	814	39.67
Middle school	925	45.08
High school and above	313	15.25
Employment		
Unemployed	797	38.84
Employed	1254	61.11
Income (CNY/Month/person)		
150 or less	936	45.61
151–300	475	23.15
300 or greater	575	28.02
Marital Status		
Never married	145	7.07
Married/cohabiting	1867	90.98
Divorced/separated/widowed	40	1.95
Religion		
Yes	205	9.99
No	1847	90.01
Depression ^b^		
No	1397	68.08
Yes	655	31.92
Anxiety ^b^		
No	1569	76.46
Yes	483	23.54
Alcohol abuse		
No	1844	89.86
Yes	208	10.14

*Abbreviation*: *CNY* Chinese Yuan

^a^ Some percentages don’t add up to 100 due to missing values

^b^ Here it means screen positive for the symptoms of depression/anxiety

**Table 2:** Percentage of respondents assigning each category to describe the primary cause of the symptoms presented in the vignette ^a^

Primary cause assigned	Depression	Anxiety	Alcohol abuse
Physical problem	38.5 (36.4, 40.6)	32.7 (30.7, 34.7)	18.5 (16.8, 20.2)
Mental problem	**57.6 (55.5, 59.7)**	**59. 8 (57.7, 61.9)**	**75.0 (73.1, 76.9)**
Others	3.9 (3.1, 4.7)	7.5 (6.3, 8.6)	6.5 (5.4, 7.6)

Characters in bold means statistical significance at *p* = 0.05

^a^ Categories were selected by participants in response to the following question: “What do you think is the primary cause of this problem?”

**Table 3:** Percentage of respondents assigning each category to label the symptoms presented in the vignettes ^a^

Category assigned	Depression	Anxiety	Alcohol abuse
	Percentage(95%CI)		
Physical weakness	17.6 (16.0, 19.2)	8.7 (7.5, 9.9)	3.1 (2.3, 3.8)
Acute anxiety attack(Panic attack)	NA	**15.5** (**13.9, 17.1**)	NA
Neurasthenia	10.8 (9.5, 12.1)	10.0 (8.7, 11.3)	5.8 (4.8, 6.8)
Depression	**16.1 (14.5, 17.7)**	7.6 (6.5, 8.7)	3.5 (2.7, 4.3)
Mania	2.0 (1.4, 2.6)	2.2 (1.6, 2.8)	1.1 (0.6, 1.5)
Obsessive-compulsive disorder	0.6 (0.3, 0.9)	1.6 (1.1, 2.2)	0.7 (0.3, 1.0)
Schizophrenia	9.9 (8.6, 11.2)	9.1 (7.9, 10.4)	7.2 (6.1, 8.3)
Alcohol-related mental disorders	NA	NA	**58.2** (**56.1,60.4**)
Others	2.0 (1.4, 2.6)	3.0 (2.3, 3.8)	0.8 (0.4, 1.2)
Unknown	40.9 (38.8, 43.0)	42.3 (40.1, 44.4)	19.6 (17.9, 21.4)

NA means there is no such answer set up for this question of the vignette

Characters in bold means statistical significance at *p* = 0.05

^a^ Categories were selected by participants in response to the following question: “What do you think is wrong with [name]?” Please fill in the item (choose only one) that you think best describes his [her] problem

**Table 4:** Univariate and multivariate analyses of the influence of socio-demographics, and mental disorders on the correct recognition of the three vignettes (*N* = 2052)

Variables	Depression		Anxiety		Alcohol abuse	
	Crude OR	Adjusted OR	Crude OR	Adjusted OR	Crude OR	Adjusted OR
Gender						
Male	ref	ref	ref	ref	ref	ref
Female	0.85(0.66, 1.08)	0.99(0.73, 1.34)	1.08(0.83, 1.40)	1.20(0.87, 1.65)	1.14(0.96, 1.36)	**1.31(1.06, 1.61)**
Age (year)						
18–25	ref	ref	ref	ref	ref	ref
26–35	0.77(0.53, 1.12)	1.07(0.68, 1.68)	0.71(0.47, 1.07)	0.76(0.47, 1.23)	0.90(0.64, 1.26)	1.00(0.68, 1.47)
36–45	**0.37(0.26, 0.53)**	0.67(0.42, 1.07)	**0.45(0.31, 0.66)**	0.67(0.41, 1.09)	0.92(0.68, 1.24)	1.07(0.75, 1.56)
46–60	**0.22(0.15, 0.33)**	**0.41(0.26, 0.67)**	**0.29(0.20, 0.43)**	**0.45(0.28, 0.74)**	0.83(0.62, 1.12)	1.03(0.71, 1.48)
Education						
Primary school or less	ref	ref	ref	ref	ref	ref
Secondary school	**3.44(2.43, 4.86)**	**2.65(1.84, 3.82)**	**2.61(1.86, 3.67)**	**2.25(1.57, 3.24)**	**1.22(1.01, 1.47)**	1.20(0.98, 1.48)
High school and above	**8.75(5.98, 12.81)**	**6.22(4.05, 9.52)**	**5.51(3.77, 8.07)**	**4.53(2.93, 7.00)**	**1.59(1.22, 2.07)**	**1.62(1.20, 2.20)**
Employment						
Unemployed	ref	ref	ref	ref	ref	ref
Employed	**0.72(0.55, 0.93)**	0.75(0.55, 1.01)	0.91(0.70, 1.19)	0.86(0.63, 1.17)	0.91(0.76, 1.09)	0.88(0.72, 1.08)
Income (RMB/Month)						
150 or less	ref	ref	ref	ref	ref	ref
151–300	**1.45(1.07, 1.98)**	1.27(0.91, 1.76)	1.22(0.88, 1.68)	1.04(0.74, 1.46)	1.03(0.82, 1.28)	0.97(0.77, 1.21)
301 or greater	**1.51(1.13, 2.01)**	1.24(0.91, 1.70)	1.08(0.79, 1.47)	0.89(0.64, 1.25)	**1.34(1.09, 1.65)**	1.23(0.99, 1.53)
Marital Status*						
Married/cohabited	ref	ref	ref	ref	ref	ref
Never married	**2.32(1.58, 3.42)**	1.19(0.71, 1.98)	1.33(0.84, 2.10)	076(0.43, 1.35)	1.15(0.82, 1.61)	1.13(0.75, 1.70)
Divorced/separated	0.49(0.15, 1.62)	1.01(0.29, 3.47)	0.17(0.02, 1.25)	0.31(0.04, 2.31)	0.71(0.38, 1.35)	0.90(0.46, 1.75)
Religion						
No	ref	ref	ref	ref	ref	ref
Yes	0.90(0.61, 1.34)	0.72(0.47, 1.11)	1.21(0.77, 1.91)	1.09(0.68, 1.77)	1.09(0.82, 1.46)	1.05(0.78, 1.43)
Depression						
No	ref	ref	ref	ref	ref	ref
Yes	0.78(0.59, 1.02)	0.85(0.60, 1.22)	0.88(0.66, 1.16)	0.90(0.63, 1.30)	0.85(0.71, 1.03)	0.89(0.70, 1.12)
Anxiety						
No	ref	ref	ref	ref	ref	ref
Yes	**0.64(0.47, 0.88)**	0.81(0.54, 1.22)	0.88(0.66, 1.16)	0.91(0.60, 1.37)	0.90(0.74, 1.11)	1.08(0.83, 1.39)
Alcohol abuse						
No	ref	ref	ref	ref	ref	ref
Yes	0.92(0.61, 1.38)	0.78(0.49, 1.26)	0.90(0.58, 1.40)	0.93(0.56, 1.53)	0.95(0.71, 1.26)	1.06(0.77, 1.45)

Characters in bold means statistical significance at *p* = 0.05

## Results

### Socio-demographics and clinical characteristics of the sample

Socio-demographics and prevalence of disorders are shown in Table 1. Most of the participants are married (91 %), non-religious (90 %), and have an educational level of middle school or below (85 %). 61 % of the sample is employed, and over two-thirds have a monthly income of lower than 300 RMB (47.4 USD). Over half (56 %) are female. Age of the participants ranges from 18 to 60, with a median of 42. Among the three mental disorders, the positive screening rate for the symptoms of depression is the highest, at 32 %, followed by anxiety (24 %) and alcohol abuse (10 %).

### Recognition of three vignettes: correctly attributing it as being a mental disorder

The proportion of respondents recognizing each vignette as reflecting mental problems were high, with a range of 57.6–75.0 %. As presented in Table 2, the alcohol abuse vignette was more frequently attributed as a mental problem than the depression and anxiety vignettes. Three-quarters of respondents correctly attributed the vignette of alcohol abuse as a mental problem, while less than 60 % of the respondents correctly attributed the vignettes of depression and anxiety as mental problems. In fact, nearly one-third of respondents misattributed the vignettes of depression and anxiety as physical problems, while physical attribution of the alcohol abuse vignette was less than 20 %.

### Recognition of three vignettes: Correctly labeling the disorder

Findings regarding disease labeling are summarized in Table 3, which shows the percentage of respondents choosing each category to label the symptoms depicted in the vignettes. Only 4.0 % of the respondents correctly labelled all three vignettes. Correct labeling was highest for alcohol abuse, with over half (58.2 %) correctly identifying the vignette as an alcohol-related mental disorder and nearly one-fifth labeled it as “I don’t know”. 16.1 % of respondents correctly labeled depression and 15.5 % correctly labeled anxiety; up to 40 % responded ‘I don’t know’. The depression vignette was most frequently mislabeled as “physical weakness”, while “neurasthenia” and “schizophrenia” were the terms most often used for the anxiety and alcohol abuse vignette, respectively.

### Factors predicting the correct recognition of three vignettes

Univariate logistic regression was first used to examine whether demographic characteristics (gender, age, education, employment, income, marital status, and religion), as well as experience of mental disorders (depression, anxiety, and alcohol abuse) influenced the correct recognition of three vignettes. For all three vignettes, education was the common factor significantly positively associated with recognition, with an odds ratio (OR) of 1.22-8.75 for higher education. Higher income was significantly associated with increased likelihood of recognizing depression and alcohol abuse, while being older was negatively associated with correct recognition of depression and anxiety. Additionally, there were three factors that were only significantly associated with recognition of depression: employment, marital status, and anxiety. A subsequent multivariate logistic regression with all the factors above included found that only education remains as a significant predicting factor for recognition of all three vignettes, with an OR ranging from 1.2 to 6.2. Age was a significant predictor for correct of recognition of depression and anxiety, with those aged 46–60 less likely to correctly recognize depression and anxiety than those aged 18–25. Furthermore, being female was an independent predictor of recognition of alcohol abuse. Results for both univariate logistic regression and multivariate logistic regression are shown in Table 4.

## Discussion

This is the first study to our knowledge to assess the recognition of depression, anxiety, and alcohol abuse, as well as it predictive factors in a representative Chinese rural sample. Our findings showed that there was great variability in the correct labeling rate for the three vignettes. The highest labeling rate was for the alcohol abuse vignette, with nearly 60 % correctly identifying the vignette as alcohol-related mental disorder, whereas less than 20 % of respondents correctly named depression and anxiety. The alcohol abuse vignette was more frequently attributed as a mental problem than the depression vignette and anxiety vignette, although over half of respondents recognized each vignette as a mental problem. Higher education is the common and also strongest factor positively predicting the recognition of all three vignettes. Beyond that, being female is an independent predictor of correct recognition of alcohol abuse, while recognition of depression and anxiety were positively predicted by younger age. This information not only helps us understand rural residents’ knowledge of common mental disorders, but also holds promise for furthering our understanding of why people with mental disorders do not seek help, providing guidance for improving the under-utilization of mental health services in the rural areas of China.

The highest recognition rate was for the alcohol abuse vignette, with 75 % recognizing it as a mental problem and 58.2 % correctly naming the disorder, which is not surprising given the high consumption of alcohol in rural areas and the frequent coverage of alcohol abuse related problems in the mass media [57, 58]. However, the much lower recognition rate for depression and anxiety was somewhat unexpected and warrants concern, with over 40 % of respondents unable to correctly recognize the two vignettes as mental problems. The correct labeling of the depression vignette was only 16.1 % in the present study, much lower than that in developed countries such as Australia (75 %) [28] and the US (58 %) [29], and also lower than that in urban areas in China (35 %) [30]. The correct labeling of the anxiety vignette was 15.5 %, still much lower than that in Australia (47.7 %) [17] but comparable to that in the US (16.6 %) [10]. The reason may be that there have been greater efforts invested into improving the public’s mental health literacy in developed countries. For instance in Australia, considerable investment of both time and funding was invested towards the national Beyond Blue campaign aimed at increase the recognition and understanding of mental health disorders among the general population. This information suggests that in developing countries like China, there is still much room for improvement in the recognition of common mental disorders such as depression and anxiety which may be highly prevalent yet long neglected. As the initial step towards help-seeking behaviors, strengthening the recognition of such mental disorders will greatly improve the utilization of mental health services in the rural areas of China [10, 18].

For the depression and anxiety vignettes, apart from the correct labels, the top three mislabels were physical weakness, neurasthenia, and schizophrenia, with less than 3 % of respondents mislabeling them as mania or obsessive-compulsive disorder (OCD). This reflects the fact that many people may have heard of some mental disorders such as depression, anxiety, neurasthenia, and schizophrenia more often than other disorders like mania and OCD. On the other hand, they may also have difficulty in identifying each specific mental disorder and distinguishing it from other mental disorders. Furthermore, there is also great confusion between mental disorders and physical problems among this sample, considering the relatively higher proportions of respondents labeling the two vignettes as “physical weakness” (8.7–17.6 %) and “neurasthenia” (10.0–10.8 %), as well as attributing them to “physical problems” (32.7–38.5 %). The mislabeling and misattribution of depression and anxiety symptoms corresponds to the prevalent belief that depressive and anxious states do not refer to mental disorders but rather normal physical reactions that need no special treatment [10, 17, 28, 34, 59]. Another possibility may be related to the somatic manifestation of mental symptoms in Chinese culture where a somatic illness label, such as neurasthenia is more acceptable, and relieved of the stigma, fear, guilt, or ambivalence associated with mental complaints [60]. All this information points to the need to build on public knowledge of a range of mental disorders, to increase their ability in differentiating mental disorders from physical problems as well as to decrease the social stigma towards mental illness.

Regarding the predictive factors, higher education is associated with an increased likelihood of recognizing all three disorders, which is consistent with previous research showing that those who are more educated are better at correctly recognizing mental disorders [10, 30]. These findings suggest that public campaigns and education may be cost-effective ways to improve the recognition of common mental disorders in an effort to increase treatment-seeking behaviors of rural Chinese residents. The negative association between older age and recognition of depression and anxiety reflects the results of other studies suggesting younger age as positively influencing recognition of mental disorders [10, 30, 35, 36]. One possible reason may be that young people are more frequently exposed to information related to depression and anxiety through a variety of media avenues such as TV, mobile phone messages, the internet and so on, while older people in rural areas have limited access to these media and thus get less information. Female gender is an independent predictor of correct recognition of alcohol abuse, which is not surprising given that alcohol use has always been seen as a “man’s problem” in Chinese culture [61, 62]. In China, drinking and smoking are seen as social habits and are very common among men in social situations, especially in the rural areas. As a result, females are more likely to correctly recognize alcohol abuse than men who treated it as a normal social habit. These findings are useful to consider in an effort to improve the recognition of mental disorders through implementing different interventions targeted for different demographic sectors.

There are two primary limitations of the present study that may provide implications and guidance for future research. First, only three vignettes were presented here to assess the residents’ recognition of depression, anxiety, and alcohol abuse, which may not be representative of all common mental disorders. In the future, it may be worthwhile to provide a wide range of vignettes that are more representative of the most common mental disorders, as well as different stimuli for each disorder. Second, we only asked two questions assessing the respondent’s recognition of the disorders, and did not assess additional factors, such as their intentions and preferences for treatment when needed and where they should go for help. The assessment of the association between increased recognition of mental disorders and increased treatment-seeking was thus lacking in the present study, although this has been proved by a wealth of research [6, 17–19, 22, 23]. Future research may benefit from adding more information on respondents’ preferences for treatment in the vignettes, as well as local doctors’ ability to recognize and treat such mental disorders.

## Conclusions

Data from the current study from 2052 rural residents in Liuyang county of China suggest that there is potential for gains in recognition of mental disorders especially depression and anxiety, which will improve access to care as well as general public mental health literacy. Recognition of common mental disorders could be improved through general public campaign and education, while paying attention to the unique demographic factors related to literacy for each specific disorder in order to implement targeted education.

## Ethics and consent to participate statement

Ethics approval was granted by the Ethics Review Committee of the School of Public Health of Central South University. Each participant has provided written informed consent before participating in the study.

## Consent to publish statements

Not applicable.

## Availability of data and materials

All data and materials related to the study can be obtained through contacting the first author at youxiang8864@163.com.

## Additional file

Additional file 1: English language copy of the questionnaire. (DOCX 29 kb)
