# Supplementary material for: Recognition of depression, anxiety, and alcohol abuse in a Chinese rural sample: a cross-sectional study
Source: BMC Psychiatry. 2016 Apr 6;16:93. doi: 10.1186/s12888-016-0802-0 (PMC4822293; doi:10.1186/s12888-016-0802-0)
Supplement: Additional file 1: — English language copy of the questionnaire. (DOCX 29 kb) [file 12888_2016_802_MOESM1_ESM.docx]

**Assessment of the mental health of the rural population**

Date: ____/____/______ Subject ID: ________

Location: ____**:**___ _

Start Time: ____**:**___ _ End Time: ____**:**___ _

Administrator notes: ________________________________

**Thank you for agreeing to participate in this study. The information you provide will help us to better understand the mental health status and mental health literacy of the rural population. Please answer the following questions as completely as you are able. You reserve the right not to answer questions if you choose. What is most important is that you tell us what you are experiencing, not what you think we want to hear. The information you provide here will be seen only by authorized research staff—none of your answers will be seen by anyone else you may know. The interview may take approximately 30-40 minutes. We appreciate your cooperation in this study.**

1. **SOCIODEMOGRAPHICS**
2. Name: ______
3. Gender: 1) Male 2) Female
4. What is your date of birth? ____/____/______ (Year/Month/Day)
5. Your marital status:

1) Single, never married 2) Married 3) Steady partner 4) Divorced 5) Widowed

6) Separated 7) Refuse to answer

1. Your education:

1) Illiterate 2) Primary school and below 3) Middle school 4) High school

5) College 6) Postgraduate and above

1. Your current occupation:

1) Employed 2) Unemployed

1. Your family monthly income per person: ________CNY/month/person
2. Do you have any religion? 1) Yes ________ 2) No

**B. PHQ-9**

| In the last 2 weeks, how often have you been bothered with any of the following problems? | Not at all | Several days | More than half of the days | Nearly every day |
| --- | --- | --- | --- | --- |
| 1. Little interest or pleasure in doing things? | 0 | 1 | 2 | 3 |
| 1. Feeling down, depressed, or hopeless? | 0 | 1 | 2 | 3 |
| 1. Trouble falling or staying asleep or sleeping too much? | 0 | 1 | 2 | 3 |
| 1. Feeling tired or having little energy? | 0 | 1 | 2 | 3 |
| 1. Poor appetite or overeat? | 0 | 1 | 2 | 3 |
| 1. Feeling bad about yourself or that you are a failure or have let yourself or your family down? | 0 | 1 | 2 | 3 |
| 1. Trouble concentrating on things, such as reading the newspaper or watching television? | 0 | 1 | 2 | 3 |
| 1. Moving or speaking so slowly that other people could have noticed? Or the opposite – Are you so fidgety or restless that you have been moving around a lot more than usual? | 0 | 1 | 2 | 3 |
| 1. Thoughts that you be better off dead, or of hurting yourself? | 0 | 1 | 2 | 3 |

**C. PHQ GAD-7**

| In the last 2 weeks, how oft have you been bothered with any of the following problems? | Not at all | Several days | More than half of the days | Nearly every day |
| --- | --- | --- | --- | --- |
| 1. Feeling nervous, anxious or on edge? | 0 | 1 | 2 | 3 |
| 1. Not being able to stop or control worrying? | 0 | 1 | 2 | 3 |
| 1. Worrying too much about different things? | 0 | 1 | 2 | 3 |
| 1. Trouble relaxing? | 0 | 1 | 2 | 3 |
| 1. Being so restless that it is hard to sit still? | 0 | 1 | 2 | 3 |
| 1. Becoming easily annoyed or irritable? | 0 | 1 | 2 | 3 |
| 1. Feeling afraid as if something awful might happen? | 0 | 1 | 2 | 3 |
| 1. Moving or speaking so slowly that other people could have noticed? Or the opposite – Are you so fidgety or restless that you have been moving around a lot more than usual? | 0 | 1 | 2 | 3 |
| 1. Thoughts that you be better off dead, or of hurting yourself? | 0 | 1 | 2 | 3 |

**D. AUDIT**

Now I am going to ask you some questions about use of alcoholic beverages in the past 1 year.

Approx. size of one drink (spirits – 40 ml, wine- 140 ml, Liquor – 125 ml, Wine – 60 ml, Beer – 330ml, country liquor/arrack/sugar cane spirit – 30 ml); assumes 12% ethanol for wine, 5% ethanol for beer & 40% ethanol for spirits.

1. How often do you have a drink containing alcohol?

0=Never 1=Monthly or less 2=2-4 times a month

3=2-3 times a week 4=4 or more times a week

1. If yes, how many standard drinks containing alcohol do you have on a typical day when drinking?

0= 1 or 2 1= 3 or 4 2= 5 or 6 3= 7 to 9 4= 10 or more

1. How often do you have six or more drinks on one occasion?

0= Never 1= Less than Monthly 2= Monthly 3= Weekly 4=Daily or almost daily

1. During the past year, how often have you found that you were not able to stop drinking once you had started?

0= Never 1= Less than Monthly 2= Monthly 3= Weekly 4=Daily or almost daily

1. During the past year, how often have you failed to do what was normally expected of you because of drinking?

0= Never 1= Less than Monthly 2= Monthly 3= Weekly 4=Daily or almost daily

1. During the past year, how often have you needed a drink in the morning to get yourself going after a heavy drinking session?

0= Never 1= Less than Monthly 2= Monthly 3= Weekly 4=Daily or almost daily

1. During the past year, how often have you had a feeling of guilt or remorse after drinking?

0= Never 1= Less than Monthly 2= Monthly 3= Weekly 4=Daily or almost daily

1. During the past year, have you been unable to remember what happened the night before because you had been drinking?

0= Never 1= Less than Monthly 2= Monthly 3= Weekly 4=Daily or almost daily

1. Have you or someone else been injured as a result of your drinking?

0= No 1= Yes, but not in the past year 2= Yes, during the past year

1. Has a relative or friend, doctor or other health worker been concerned about your drinking or suggested you cut down?

0= No 1= Yes, but not in the past year 2= Yes, during the past year

**E. Vignettes**

Vignette 1

Mr. Wang is 30 years old. He has been feeling unusually sad and miserable for the last few weeks. Even though he is tired all the time, he has trouble sleeping nearly every night. Mr. Wang does not feel like eating and has lost weight. He cannot keep his mind on his work and puts off making decisions. Even day-to-day tasks seem too much for him. This has come to the attention of his boss, who is concerned about Mr. Wang’s lowered productivity.

1. What do you think is the primary cause of this problem? Please fill in the item (choose only one) that you think best explains his problem.
2. Physical problem
3. Mental problem
4. Possession by evil spirits
5. Over fatigue
6. Others
7. What do you think is wrong with Mr. Wang? Please fill in the item (choose only one) that you think best describes his problem.
8. Physical weakness
9. Neurasthenia
10. Depression
11. Mania
12. Obsessive-compulsive disorder
13. Schizophrenia
14. Others
15. Unknown

Vignette 2

Mr. Li is 25 years old and he is an engineer. For one year, he has been under great pressure due to work stress and a strained relationship with his boss. Two months ago, during a very important company meeting, he suddenly had increased heart rate, chest pain, and shortness of breath, accompanied with a lot of sweating and numbing limbs. Mr. Li was horrified, fearing that he was dying. He was immediately sent to the emergency room of a hospital nearby, but no abnormality was found during the physical check. Half an hour later, all symptoms were relieved. Mr. Li was conscious throughout the whole attack. From then on, Mr. Li got similar attacks almost every week without any obvious physical damage during the interval of attack, but he was very scared of experiencing more attacks. There was no obvious inducement for every attack, nor was it related to some certain occasion. Every time when Mr. Li got an attack, he would be sent to a hospital by an ambulance, but no abnormality was found.

1. What do you think is the primary cause of this problem? Please fill in the item (choose only one) that you think best explains his problem.
2. Physical problem
3. Mental problem
4. Possession by evil spirits
5. Over fatigue
6. Others
7. What do you think is wrong with Mr. Li? Please fill in the item (choose only one) that you think best describes his problem.
8. Physical weakness
9. Acute anxiety attack(Panic attack)
10. Neurasthenia
11. Depression
12. Mania
13. Obsessive-compulsive disorder
14. Schizophrenia
15. Others
16. Unknown

Vignette 3

Mr. Wang is an old worker in a nearby factory. He is a kind person who works very hard and has a happy family. However, Mr. Wang has a special hobby of drinking alcohol. For the past 20 years he has been drinking alcohol on a daily basis. Every morning when he wakes up the first thing he does is to look everywhere for alcohol, otherwise he will feel uneasy and cannot concentrate on work all day. Recently, his family has found that Mr. Wang is becoming strange. He is always suspicious, suspecting his wife is cheating on him, suspecting somebody is stealing things from his house. Every day he is so paranoid that there is barely any peace in his family. Finally, he is sent to the doctor by his family.

1. What do you think is the primary cause of this problem? Please fill in the item (choose only one) that you think best explains his problem.

① Physical problem

② Mental problem

③ Possession by evil spirits

④ Over fatigue

⑤ Others

1. What do you think is wrong with Mr. Wang? Please fill in the item (choose only one) that you think best describes his problem.
2. Physical weakness
3. Neurasthenia
4. Depression
5. Mania
6. Obsessive-compulsive disorder
7. Schizophrenia
8. Alcohol-related mental disorders
9. Others
10. Unknown
